# A Correlation Between Migraine and Endometriosis and Its Clinical Implications—A Systematic Literature Review

**DOI:** 10.3390/jcm14082744

**Published:** 2025-04-16

**Authors:** Ewelina Lechowicz, Aleksander Łaciński, Antonina Smulska, Olga Grodzka, Izabela Domitrz

**Affiliations:** 1 Department of Neurology, Faculty of Medicine and Dentistry, Medical University of Warsaw, 02-091 Warsaw, Polands090558@student.wum.edu.pl (A.S.);; 2 Doctoral School, Medical University of Warsaw, 02-091 Warsaw, Poland

**Keywords:** endometriosis, endometrium, menstruation, migraine, primary headache disorders, uterus

## Abstract

**Background/Objectives:** Migraine and endometriosis are two diseases that are associated with women. Endometriosis is a condition exclusively affecting the female population as it affects the female genital tract, while migraine is a primary headache disorder having the highest prevalence in women of reproductive age. Although, undoubtedly, they are two distinct disorders, some correlations have been suggested considering the epidemiological similarities. **Methods:** This systematic review aimed to analyze the putative links between those two diseases. Two databases were searched in accordance with the PRISMA guidelines, which led to the inclusion of 28 of the most appropriate studies. The review was registered in PROSPERO. **Results:** A comprehensive analysis of the existing literature allowed us to distinguish six different aspects: (i) the prevalence of migraine in the course of endometriosis, in general, (ii) when comparing endometriosis patients to healthy individuals, (iii) the relation between different migraine types and endometriosis, (iv) pain symptoms in patients with endometriosis and migraine, and finally, (v) molecular and (vi) genetic bases of the suspected correlation. **Conclusions:** Although not all results are definitely apparent, the results showed a higher prevalence of endometriosis and migraine together than both diseases separately. More precisely, chronic migraine was demonstrated to be the most possibly linked to endometriosis. Moreover, pain symptoms were usually more evident in patients suffering from both diseases at the time. Finally, some suggestions were presented due to this comorbidity’s molecular and genetic bases; however, the literature, especially on this topic, is lacking.

## 1. Introduction

### 1.1. Background About Migraine

Migraine is a common type of primary headache disorder, ranked as the third-highest cause of disabilities worldwide amongst both sexes under the age of 50 [1]. Over 1 billion people world-wide are affected by migraines, with women suffering more frequently than men [2]. Moreover, according to the Global Burden of Disease Study 2021, disability-adjusted life years (DALYs) increased from 1990 to 2021 by over 50%, showing that migraine is a very up-to-date problem causing disability among young, professionally active people [2].

According to the International Classification of Headache Disorders, 3rd edition (ICHD-3), migraine manifests as reoccurring attacks usually characterized by unilateral, pulsating pain of moderate or severe intensity that is aggravated by routine physical activity. These attacks are accompanied by nausea and/or phonophobia and photophobia. Attacks tend to last 4–72 h; however, they can last less if treated successfully [3].

Migraine is divided into chronic and episodic subtypes. For migraine to be considered chronic, patients must experience a headache at least 15 days per month for more than 3 months where, during at least eight days, the migraine criteria must be fulfilled [3]. Furthermore, the two main types of episodic migraines are migraine without aura and migraine with aura, where an aura is the differentiating feature [3,4]. An aura is a fully reversible phenomenon, and might manifest as visual, sensory, speech, motor, or other, more rare, symptoms. Occurring aura symptoms spread gradually for over 5 min and last for up to an hour. Within 60 min from the beginning of the aura, the headache ought to occur; however, cases of an aura not followed by a headache have also been reported [3,5]. Additionally, some patients experience a set of prodromal symptoms that occur before the aura itself. That might include fatigue, apathy, neck stiffness, nausea, appetite disorders, blurred vision, yawning, and pallor, among many more [6,7].

Despite the wide spread of migraine, it is still a severely mis- and underdiagnosed condition [8,9]. Moreover, studies have shown that the costs to patients with a history of migraine misdiagnosis were higher than those with correctly diagnosed migraine, demonstrating the importance of proper diagnosis not only for individual well-being but for general health-related economics [8]. According to data reported by neurologists and primary-care physicians themselves, worldwide, up to 30% of primary-care physicians and 34% of neurological consultations for headaches turn out to be migraine [10].

### 1.2. Background About Endometriosis

Endometriosis is considered an inflammatory disorder of the female reproductive system characterized by the growth of endometrial tissue outside of the uterine cavity. This tissue, even if placed outside of its proper location, is subject to all the changes related to the menstrual cycle [11]. According to the World Health Organization (WHO) data, endometriosis affects up to 10% of reproductive-age women, which makes around 190 million people globally [11,12].

The location of the lesion determines the type of endometriosis. The most common differentiated types include superficial endometriosis (found in the pelvic cavity only), endometrioma (found in the ovaries), and deep endometriosis (affecting the rectovaginal septum, bladder, or intestines) [13]. Cases of endometrial lesions found outside of the pelvic cavity have also been reported; the abdominal and chest cavities and the diaphragm are found to be possible loci of the disease [14,15]. A chronic state of inflammation that underlies this condition leads to scarring covering the affected area, resulting in adhesions and fibrosis [16,17].

The condition consists of a wide variety of symptoms, individually variable; however, the most prevalent include dysmenorrhea, dyspareunia, dysuria, menorrhagia, intermenstrual bleeding, pelvic pain, bloating, nausea, fatigue, and, as a result, often infertility [18]. Due to the deliberating character of the disorder, most patients develop anxiety and depression, adding a set of psychological symptoms that coexist with the physical ones. Four stages of endometriosis can be distinguished, according to the American Society of Reproductive Medicine (ASRM) guidelines, which correspond to minimal, mild, moderate, and severe endometriosis (I, II, III, and IV, respectively) [19].

Screening tools used to diagnose patients might vary, depending on the localization of pain. First and foremost, a detailed history of menstrual symptoms and chronic pelvic pain allows the physician to suspect endometriosis. Histopathological analysis of laparoscopic visualization is required to confirm the diagnosis. Pap smears, urinalysis, or endocervical swabs might be necessary to rule out other conditions. Ultrasonography (USG) is used to visualize pelvic masses; transvaginal USG is used for the uterine cavity and the endometrium; whereas transabdominal USG is used for masses on the ovaries [12,20]. The lack of biochemical markers available as definitive clinical diagnostic tools leads to patients being severely underdiagnosed [21,22]. On average, patients take several years to receive a final diagnosis, which requires laparoscopic surgery for confirmation [22,23].

### 1.3. The Aim of the Review

Since endometriosis affects women of reproductive age, while migraine is a condition that appears in this specific population most frequently, the possibility of some links between those two disorders has been hypothesized. The association has been considered controversial; however, in a recent meta-analysis, a significant correlation between those two has been proven (OR 1.56; 95%CI: 1.21, 1.90), based on 802 included high-quality articles and over 287,000 participants [24]. Moreover, a narrative review performed in 2022 demonstrated that both migraine and endometriosis share at least three overlapping gene polymorphisms regarding sex hormone genes, more precisely, estrogen receptor genes [25]. Thus, the association has been suggested to exist both from a clinical and a pathogenetic point of view. This systematic review aims to summarize the current knowledge on the correlation between migraine and endometriosis from different points of view, and attempts to confirm or deny a hypothesis about a putative link between those two disorders.

## 2. Materials and Methods

The systematic review was conducted according to the Preferred Reporting Items for Systematic Reviews and Meta-Analyses (PRISMA 2020) guidelines [1]. Two databases, the PubMed Database and the Embase Database, were screened with a search strategy as follows: (migraine) and (endometriosis). The study protocol was registered in PROSPERO (ID: CRD42024607296).

### 2.1. Inclusion and Exclusion Criteria

The inclusion criteria covered original articles such as randomized controlled trials and observational studies (including cohort, cross-sectional, and case-control studies). To be considered, the topic of the analyzing paper had to be related to the review title and address the issue of migraine–endometriosis comorbidity. Only studies with full text written in English were considered.

Criteria for exclusion included article types such as reviews, meta-analyses, case reports or case series, commentaries, editorials, and conference abstracts were excluded. Studies that did not cover the correlation between migraine and endometriosis were also removed. Finally, articles published in languages other than English were not allowed.

### 2.2. Data Extraction

In this work, we systematically extracted data based on predefined variables and outcomes relevant to the scope of the review. All variables for which data were sought included, but were not limited to, demographic characteristics, co-existing symptoms and disorders, disease characteristics, molecular bases issues, and genetic insights. Extracted data were summarized briefly in tables following each paragraph. This structured approach allowed for consistent and comprehensive data collection across all included studies.

### 2.3. Risk of Bias Assessment

A narrative assessment of the risk of bias was conducted for all potentially eligible studies. Two authors (E.L. and A.Ł.) independently evaluated the methodological quality of each study, with discrepancies resolved through discussion and verification by a third author (O.G.). Although no standardized tool was applied, all considered studies were deemed to possess acceptable quality in terms of key characteristics, such as clarity of research aims, appropriateness of methodology, transparency in reporting, and relevance of outcomes. As a result, all potentially eligible articles were assessed as methodologically sound and were included in the review. Being aware of the potential limitations of this approach, we have mentioned it with potential bias from the included studies at the end of the review in the appropriate section.

## 3. Findings

**Figure 1:**
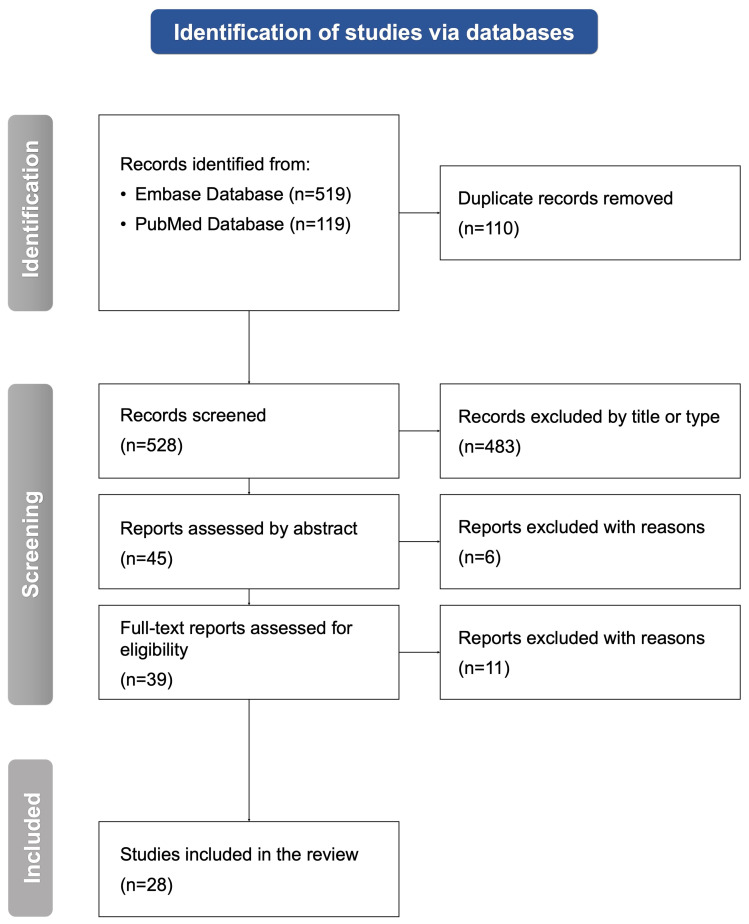
A flowchart presenting the selection process performed in accordance with the Preferred Reporting Items for Systematic Reviews and Meta-Analyses (PRISMA 2020) guidelines. n, number of studies.

Two authors (E.L. and A.Ł.) conducted a selection independently. Any discrepancies have been additionally analyzed and re-assessed with final verification by the third author (O.G.) to avoid bias. The selection process (Figure 1) started with the identification of 638 articles from two screened databases: the PubMed database (119 records) and the Embase database (519 records). Databases were accessed for reproducibility on 27 October 2024. The search strategy for both databases was the same and looked as follows: (migraine) AND (endometriosis). After the removal of duplicates, the remaining 528 records were screened. Four hundred eighty-three items were then excluded due to inappropriate title or type. A further search through 45 reports has led to removing an additional six items by abstract. Finally, the full-text analysis resulted in the exclusion of eleven articles. Eventually, 28 studies were chosen for inclusion in the systematic review, and are comprehensively analyzed below.

### 3.1. The General Prevalence of Migraine in the Course of Endometriosis

Research on the correlation between endometriosis and migraine has been a matter of concern and the object of studies over the years. Numerous studies have shown that migraine is a common comorbidity in patients with endometriosis. Various populations across the world have been examined for the said association. Flores-Caldera et al. [26] carried out a cross-sectional study on comorbidities reported by Iberian–American endometriosis patients from a total of 23 countries (94.6% of patients had reported having endometriosis). Migraine turned out to be one of the most common concomitant diseases (24.1%) reported by the participants. Similar numbers were shown in a prospective, direct-to-patient survey study carried out by Agarwal et al. [27]. The results showed that one of the most frequently reported comorbidities in women with self-reported endometriosis was also migraine (21.7%). In a different, observational cross-sectional study conducted by Vannuccini et al. [28] investigating Italian Caucasian women with endometriosis, it was shown that the prevalence of comorbid migraine totaled 54%; more than twice as much as in the previous two studies. However, all these studies indicate the great importance of migraine as a common coexisting condition in endometriosis patients. In a cross-sectional study by Sarria-Santamera et al. [29], data from 4055 women with endometriosis were analyzed and used to identify six clusters of women differentiated by their comorbidities in the course of endometriosis. One of the clusters (cluster six) comprised women with headaches and migraines. The said cluster had the highest incidence of women aged between 21 and 30 (14.96%), and had the highest commonness of headache/migraine (96.4%).

Information about a family history of endometriosis or migraine/concomitance of these two diseases in patients’ family members may potentially be helpful in an earlier diagnosis of endometriosis or migraine in these patients. A case-control study conducted by Metzler et al. [30] investigated the impact of migraine family history in women with histologically confirmed endometriosis. The study revealed that 33.4% of the examined patients had a positive family history of migraine (for migraine in a first-degree relative, it was 23.8%), and that a family history of endometriosis and migraine was reported in 3.5% of cases. The study showed that a positive family history of endometriosis was not associated with a positive family history of migraine (*p* = 0.910); however, a positive family history of migraine was significantly associated with a lower response of endometriosis symptoms to combined hormonal contraceptives.

Although several studies have shown that migraine is common in patients with endometriosis, a prospective study by Karp et al. [31] indicated no significant correlation between these two diseases. The study compared the prevalence rate of migraine between women with chronic pelvic pain without endometriosis and women with pathologically diagnosed endometriosis. Headaches were identified as migraine or non-migraine using the International Headache Society criteria. As a result, the lifetime prevalence of migraine, either definite or possible, in women with chronic pelvic pain was 67%; however, women with endometriosis had no increased risk of migraine compared to women without endometriosis. This study emphasizes that this issue requires further investigation. All studies covered in this section, with additional information, are summarized in Table 1.

### 3.2. Migraine Is More Common in Women with Endometriosis than in Healthy Controls

Several studies have investigated the prevalence of migraines among endometriosis patients in comparison to healthy controls. Among these is a study conducted by Ferrero et al. [32], where histologically confirmed endometriosis patients and healthy women were evaluated by a neurologist experienced in headache management. Both groups were screened for primary and secondary headaches, with the result of only migraine being significantly more frequent among women with endometriosis than healthy individuals (38.3% and 15.1%, respectively). Both migraines with and without aura were more common. A different, population-based study by Yang et al. [33] analyzed a large endometriosis patients’ cohort to search for potential comorbidity with migraine. Once again, the prevalence of diagnosed migraine among endometriosis patients in comparison to healthy controls was found to be much higher (4.9% and 2.9%, respectively, OR = 1.70). Wu et al. [34] took a similar approach to this question in their study but expanded the topic by searching for a correlation with a specific type of endometriosis, adenomyosis. Although this steps away from the main objective of this review, it is an interesting observation that they found endometriosis patients with co-occurring adenomyosis even more likely to suffer from migraines than patients with isolated endometriosis. These women also had a higher prevalence of migraines in comparison to individuals with adenomyosis only. As expected, based on the previous studies, both groups, endometriosis with adenomyosis and endometriosis without adenomyosis, were much more frequently diagnosed with migraine than healthy women. Another study focusing on this topic was one by Karamustafaoglu Balci et al. [35], which also compared migraine prevalence among women with confirmed endometriosis to those without it. Results were unsurprising, with 44.7% of endometriosis patients compared to 26.8% of controls screening positive for migraine. Sultana et al. [36], in their recent study, analyzed migraine as a concomitant disease in the population with endometriosis. Individuals with endometriosis, when compared to healthy controls, had 5.4 times greater odds of suffering from migraine. Al-Jefout et al. [37] analyzed self-reported comorbidities of endometriosis and compared them to their occurrence in healthy women. Among those comorbidities were migraines, which were reported at a rate significantly higher among women with endometriosis than in healthy controls. Similar to previously discussed studies, Swift et al. [38] also compared the prevalence of migraine in women suffering from endometriosis and healthy women, with a result of 19.8% and 13.2%, respectively.

Tietjen et al. [39] took a different approach to presumed comorbidity between migraine and endometriosis. They studied the frequency of menorrhagia and endometriosis in migraine patients compared to healthy women. Unanimously, with all previously mentioned research, results showed that women who experienced migraines in the past much more frequently reported being diagnosed with endometriosis (30% and 4%, respectively). In a different study, Tietjen et al. [40] also calculated the frequency of endometriosis among the migraine population. As seen previously, individuals with migraine suffer more frequently from endometriosis in comparison to healthy controls (22% and 9.6%, respectively). All this research consistently agrees that women with endometriosis more commonly suffer from migraines, and women with migraines are more likely to be diagnosed with endometriosis in comparison to healthy individuals. All studies covered in this section, with additional information, are summarized in Table 2.

### 3.3. Different Migraine Types in Relation to Endometriosis

In addition to demonstrating that migraine is a common comorbidity in women with endometriosis, several studies have investigated the prevalence of particular types of migraine comorbid with endometriosis, and revealed that certain types are more frequent than others. An observational pilot study by Merki-Feld et al. [41] focused on the incidence of migraine with aura in the course of endometriosis. Patients suffering from migraines and endometriosis were interviewed for the evaluation of the character of migraine based on criteria of the ICHD-3. Postmenopausal women and those with scar endometriosis or adenomyosis were excluded. Interestingly, as many as 41.5% of the women reported migraine with aura, which led to the conclusion that endometriosis is highly linked to migraine with aura.

A different prospective case-control study conducted by Pasquini et al. [42] concentrated on the division of migraine coexisting with endometriosis into pure menstrual, menstrually-related, and non-menstrual. Out of 131 women with endometriosis, 70 (53.4%) were diagnosed with migraine. Regarding the criteria of ICHD-3, these patients were classified according to the subtypes of migraine. Menstrually-related migraine (defined as occurring on day 1± of menstruation, and additionally at other times of the cycle) was reported by 45.7% of participants, while 18.6% of them had pure menstrual migraine (defined as migraine occurring exclusively on day 1± of menstruation). Finally, 35.7% were diagnosed with non-menstrual migraine. The study indicates that, according to the said division into subtypes, menstrually-related migraine is the most common subtype in women with endometriosis with concomitant migraine.

In the prior mentioned cross-sectional study by Tietjen et al. [40], which investigated the headache characteristics of women with endometriosis and migraine, it was found that 64% of the women suffering from chronic migraine compared to 36% of the women with episodic migraine were diagnosed with endometriosis, which suggests a strong correlation between chronic migraine and endometriosis. On the contrary, in an exploratory study conducted by Spierings and Padamsee [43], no statistically significant correlations were observed between the occurrence of endometriosis and the incidence of chronic versus episodic migraine (*p* = 0.17). Nonetheless, the study revealed that, in women with chronic migraine compared to those with episodic migraine, the prevalence of menstrual-cycle disorders in general (41.2 vs. 22.2%) was statistically significantly higher. All studies covered in this section, with additional information, are summarized in Table 3.

### 3.4. Pain Symptoms in Patients with Endometriosis and Migraine

Individuals who suffer from endometriosis and migraines often report having other pain symptoms simultaneously. In a latent class analysis by Ghiasi et al. [44], the authors identified five subgroups of women with chronic pelvic pain based on the severity, frequency, and life impact of their pain. A diagnosis of endometriosis was found to be the strongest predictor of belonging to all subgroups marked by severe pelvic pain. Although the frequency of migraines was the highest in the subgroup highly associated with endometriosis, migraines were found to be prevalent among all patients with pelvic pain, irrespective of endometriosis diagnosis frequency. The class with the highest prevalence of both migraine and endometriosis was burdened with the greatest variety of comorbid conditions, some of them related to pain symptoms, such as nonpelvic, abdominal pain, leg pain, and fibromyalgia. The authors suggested that the correlation with fibromyalgia may be explained by these patients experiencing central sensitivity syndrome. Moreover, fibromyalgia has also been found to be more prevalent among patients with endometriosis and migraine in the previously mentioned study by Tietjen et al. [38]. All current research regarding additional symptoms in the discussed comorbid patients suggests that women affected with both endometriosis and migraine experience a range of symptoms, many of them related to central sensitivity syndrome.

Apart from additional pain symptoms, women experiencing both migraines and endometriosis tend to report more severe pain associated with both conditions. Results of a study by Maitrot-Mantelet et al. [45] showed that visual analog scale (VAS) scores for chronic pelvic pain were higher among patients with endometriosis who experienced migraines. Interestingly, this research found that individuals with this comorbidity were at risk for certain endometriosis phenotypes, specifically ovarian endometrioma and deep infiltrating endometriosis, but not for superficial peritoneal endometriosis. A study on adolescents by Miller et al. [46] found that severe migraine-related pain was positively correlated with higher odds of endometriosis. Evans et al. [47] investigated comorbid symptoms in women with dysmenorrhea. The researchers specifically examined the status of endometriosis diagnosis and found that women with dysmenorrhea experience a range of associated symptoms, regardless of their endometriosis status. Moreover, the presence of migraine headaches in endometriosis patients was positively correlated with the severity of dysmenorrhea. This is yet another example of a study that supports the idea of central sensitization as the underlying cause of this comorbidity. Neumeier et al. [48] further investigated the severity of symptoms in the discussed comorbid patients. They found that these women, in comparison to patients with only endometriosis, more frequently experienced dyschezia, dysuria, and more severe dysmenorrhea onset on menarche. Finally, a prospective case-control study by Selntigia et al. [49] investigated the severity and presence of symptoms related to migraine and endometriosis in comorbid patients. Endometriosis symptoms in patients with co-occurring migraine were more severe (higher VAS scores for dysmenorrhea, dyspareunia, dyschezia, and dysuria) and more prevalent (dysmenorrhea, dyschezia, and dysuria) than in patients with endometriosis only. Similarly, migraine parameters in patients with endometriosis were worse than in patients with just migraine (higher VAS scores, more monthly migraine days, higher Headache Impact Test (HIT-6) scores). All mentioned findings underscore the increased severity of symptoms in comorbid patients.

**Figure 2:**
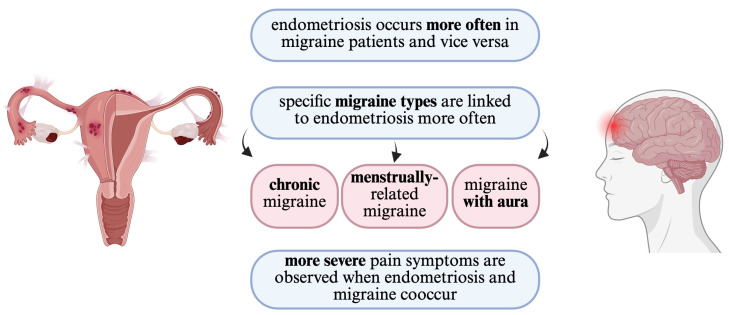
Clinical features of the correlation between migraine and endometriosis summarized graphically.

Additionally, regarding the differences between episodic migraine and chronic migraine, which have been raised previously, they also differ in case of pain symptoms. Spierings and Padamsee [43] found that dysmenorrhea was significantly more frequent in patients with chronic migraine than those suffering from an episodic type. All studies covered in this section, with additional information, are summarized in Table 4. Additionally, all features referring to the correlation between the clinical aspects of migraine and endometriosis are presented graphically in Figure 2.

### 3.5. The Putative Underlying Causes of Observed Comorbidity

As mentioned before, a few studies suggested that a potential underlying cause of migraine and endometriosis comorbidity may involve central sensitivity syndrome. This idea is supported by the symptomatic profiles of women with these comorbidities; for example, a higher prevalence of fibromyalgia and other symptoms is related to central sensitization, as further discussed in Section 4. Furthermore, research has been conducted to explore the potential molecular etiology of this comorbidity. Sasamoto et al. [50] investigated plasma proteomic profiles associated with pain subtypes in patients with endometriosis. They found a total of twenty-seven proteins, some positively and some negatively correlated with migraine in comorbid patients. Moreover, a total of twenty-eight proteins were found to correlate with distal rather than pelvic-localized pain.

Among the proteins crucial to migraine pathogenesis is calcitonin gene-related peptide (CGRP). Interestingly, one research group studied this protein in relation to the comorbidity of migraine and endometriosis. Raffaelli et al. [51] measured the plasma concentrations of CGRP throughout the menstrual cycle in four groups of women: women with migraine only, women with endometriosis only, women with both those conditions, and healthy controls. Alterations in plasma CGRP concentrations throughout the menstrual cycle differed between groups. Periovarian CGRP concentrations in comorbid patients were higher than during the perimenstrual phase, whereas the opposite pattern was observed in healthy controls. Absolute plasma concentrations of CGRP during menstruation were similar across all groups, but were significantly different during ovulation. The group of women with endometriosis and migraine had lower absolute periovulatory CGRP concentrations than healthy women. However, no significant correlation between CGRP levels and migraine or endometriosis pain frequency was found. These results point to a potential pathophysiological mechanism affecting CGRP homeostasis in comorbid patients; therefore, further research is required to draw any definite conclusions. All studies covered in this section, with additional information, are summarized in Table 5.

### 3.6. Migraine and Endometriosis from a Genetic Point of View

Finally, the common co-occurrence of endometriosis and migraine raises the question of some genetic correlation between these two diseases. A study conducted by McGrath et al. [52] focused on looking for comorbidities of endometriosis in the United Kingdom (UK) Biobank, and an exploration of the comorbid nature of these conditions using Mendelian randomization, genetic correlation, colocalization analysis, and gene-based analysis. The genetic correlation was evaluated for 74 traits (including migraine) identified in the UK comorbidity search. Migraine and 21 other traits had a significant positive genetic correlation with endometriosis, and were used in colocalization analysis. Based on the analysis performed in genome-wide association studies and pairwise analysis (GWAS-PW), one locus for migraine with suggestive evidence of having two distinct causal variants (PPA4) with endometriosis was found. The genetic correlation between migraine and endometriosis was estimated at 0.14.

A different study, carried out by Nyholt et al. [53], investigated the genetic influences underlying the comorbidity of migraine and endometriosis in a sample of 457 dizygotic and 815 monozygotic female twin pairs, for whom migraine symptom data were available, and endometriosis status had previously been determined. Twin-pair cross-tabulations revealed an increased correlation for both endometriosis and migrainous headache for monozygotic co-twins compared to dizygotic co-twins of proband twins suffering from either trait, which suggests the existence of both individual and shared genetic effects. Interestingly, no evidence was found for common environmental or non-additive genetic factors impacting either endometriosis or migraine. A significant additive genetic correlation was detected between migraine and endometriosis (rG = 0.27), whereas the observed non-shared environmental correlation between these two diseases was not significant (rE = 0.08). Furthermore, the significant bivariate heritability was assessed at 15.37%, and the non-significant non-shared environmental factors at 2.99%. That implies that common genetic influences utterly underlie the observed covariance between endometriosis and migraine. All studies covered in this section, with additional information, are summarized in Table 6.

## 4. Conclusions

Although much is already known about the diseases covered in this review, there are still some gaps. Each disease’s pathogenesis is undoubtedly the aspect that always has a significant impact on the whole management process, including diagnosis, prevention, and therapy. Therefore, insights into the molecular bases of each condition seem crucial for further achievements in the broad field of clinical medicine. To explore this concept further, some brief information about what is already known about migraine and endometriosis pathogenesis is needed.

There are two main theories about migraine pathogenesis: vascular and neural. The former was the most widely accepted until CGRP, a molecule crucial in migraine development with a role in trigeminal activity, was discovered [54]. The vascular hypothesis focuses on intracranial vasoconstriction with subsequent dilatation as causes of the two main phases of migraine: aura and pain, respectively [55]. Currently, researchers suggest that both aspects are essential, therefore enhancing neurovascular theory [56].

The pathogenetic cause of endometriosis development is also not entirely known. However, it is supposed to be associated either with the remains of the Wolffian or Mullerian duct from embryological development or with fragments of endometrium refluxing into the peritoneal cavity during menstruation [57]. In turn, refluxed progenitor and stem cells are enriched during regeneration of endometrial tissue, allowing survival in an extrauterine site. Additional activation of inflammatory pathways determines whether they are active lesions or silent fibrotic lesions [58].

To indicate some new insights into the vast topic of migraine and endometriosis pathogenesis, in the systematic review, we analyzed the links between those two diseases. Migraine turned out to be a common comorbid condition in women suffering from endometriosis from various populations. Some studies have also shown that women with endometriosis have a significantly higher prevalence of migraines. However, one study did not show a significant correlation between these diseases. Additionally, there was no association between a positive family history of endometriosis and a positive family history of migraine, but a positive family history of migraine was significantly associated with a poorer response to combined hormonal contraceptives regarding endometriosis symptoms. Considering those unclear results, there is no doubt that further research is needed.

Several studies investigated the incidence of specific migraine subtypes in patients with endometriosis. Endometriosis was shown to be highly linked to migraine with aura. Additionally, it turns out that menstrually-related migraine is the most common subtype (among pure menstrual, menstrually-related, and non-menstrual) in women with endometriosis. One study suggested a strong correlation between chronic migraine and endometriosis, although, in another one, no statistically significant correlation was observed.

Some conflicting results regarding the co-occurrence of endometriosis and migraine may be explained by several factors. Differences in study design, sample size, and diagnostic criteria for both conditions can significantly influence the findings. Additionally, variations in the populations studied—such as age, hormonal status, or presence of comorbidities—might contribute to discrepancies. Some studies may also differ in how rigorously they control for confounding variables. Finally, publication bias and limitations in self-reported data may further contribute to the observed inconsistencies. Despite these variations, the overall trend across most studies suggests a potential association between endometriosis and migraines.

Regarding the clinical presentation, individuals with both endometriosis and migraines may have more severe pain, more symptoms, and other conditions, like fibromyalgia, which may be associated with central sensitivity syndrome. Research revealed that the combination of both these diseases could result in more severe endometriosis, more frequent and more severe migraines, and worse overall disease features than either condition alone.

Finally, although the pathogenesis of this comorbidity is still unclear, some hypotheses link it to central sensitivity syndrome. Also, altered plasma protein profiles and disruptions in CGRP regulation have been proposed as potential influencing factors. From the genetic perspective, migraine and 21 other traits had a significant positive genetic correlation with endometriosis. Additionally, one locus for migraine with suggestive evidence of having two distinct variants with endometriosis was detected.

The systematic review has demonstrated that multiple variables have an impact on the putative correlation between migraine and endometriosis. In many cases, those two conditions have been found to be more prone to co-occur. Among several factors influencing this association, the underlying inflammatory process should be listed, which is crucial in the pathogenesis of both migraine and endometriosis. Another essential factor is the role of sex hormones, which are known to interfere with the course of migraine, and undoubtedly are crucial to endometriosis, as it can be called a hormone-related disease. Additionally, the included studies showed that genetics plays an essential role in both these diseases that are comorbid together.

The existing evidence suggests links between migraine and endometriosis, and the pathogenetic aspects of both diseases seem to play a significant role. Further research is required to understand the underlying mechanisms fully.

## 5. Strengths and Limitations

What adds value to this study is its comprehensive exploration of the correlation between endometriosis and migraine from multiple perspectives, including clinical, molecular, and genetic. Among the key strengths of this review is the use of a systematic approach, which ensures a structured and thorough examination of the topic. We believe the review successfully captures and synthesizes the most relevant and high-quality literature available. Additionally, the screening and risk of bias assessment were independently conducted by two reviewers, with discrepancies resolved through consultation with a third author. This methodology minimized the risk of excluding essential studies or including those that did not meet the established inclusion criteria.

This review is not without limitations. Firstly, a limited number of studies were conducted, and more research is needed in this area. Some studies included patients with self-reported endometriosis, which should be noted as a potential risk of bias. Regarding the methodology, only two databases were screened, which, although many records have been identified primarily, could lead to the absence of some studies that were unavailable in those two databases. Also, a narrative manner of the risk of bias assessment should be considered a limitation; however, two authors have assessed it independently with due diligence, including the evaluation of all crucial methodology elements, afterward being verified by the third author; thus, we believe that the risk of methodology bias has been limited. Finally, only English studies were allowed, which could possibly lead to omitting some critical studies written in other languages.

## Figures and Tables

**Table 1:** A summary of studies regarding the general prevalence of migraine in the course of endometriosis.

Ref.	Year	Population	Comparison	Findings	EM Diagnosis
Flores-Caldera et al. [26]	2021	1378 EM pts, A 33.7 (SD ± 7.2)	cross-sectional study—incidence of comorbidities in EM pts	MIG in 24.1%, PCOS in 22.2%, IBS in 21.1% of EM pts	94.6% self-reported EM
Agarwal et al. [27]	2020	84 EM pts, A 31.4 (7.1) years, 76.2% of pts Caucasian	prospective, direct-to-patient survey study—incidence of comorbidities in EM pts	depression in 34.9%, anemia in 21.7%, MIG in 21.7%, premenstrual syndrome in 16.9%, obesity in 14.5%, IBD in 10.8% of EM pts	self-reported EM
Vannuccini et al. [28]	2018	134 Italian Caucasian EM pts, A 34.8 ± 6.3	observational cross-sectional study—clinical history, pain symptoms, and systemic comorbidities	MIG in 54%, inflammatory diseases in 33%, autoimmune diseases in 18%, metabolic/endocrine diseases in 16% of EM pts; 59% PHQ (+) for a PD	OMA 37%, DIE 24%, mixed OMA plus DIE 36%, mixed OMA plus SUP 3%
Saria-Santamera et al. [29]	2023	4055 EM pts, aged between 21 and 50	cross-sectional study—clusters of women with EM based on their comorbidity	cluster 1 (less comorbidity); cluster 2 (anxiety and musculoskeletal disorders); cluster 3 (type 1 allergy or immediate hypersensitivity); cluster 4 (multiple morbidities); cluster 5 (anemia and infertility); cluster 6 (headache and MIG) has the highest frequency of patients aged between 21 and 30 (14.96%)	data extracted from medical records (from 2013 to 2017)
Metzler et al. [30]	2024	344 EM pts, A 36.2 ± 7.3 years	observational case-control study—relations between a FH for EM, MIG, depression, EMP	(+) FH for EM was not associated with a (+) FH for MIG, EMP, or depression; (+) FH for M was not associated with a (+) FH for EMP or depression; (+) FH for depression was not associated with a (+) FH for menopause; (+) FH for MIG was significantly associated with a personal manifestation of MIG	histologically confirmed EM
Karp et al. [31]	2011	81 EM pts (75%) with CPP	27 pts (25%) with CPP	NSD between MIG prevalence in EM and non-EM pts (67% and 67%); NSD between EM prevalence in MIG and non-MIG pts (75% and 75%) lifetime prevalence of definite or possible MIG was 67% of pts with CPP;	pathologically confirmed EM

(+), positive; A, mean age; CPP, chronic pelvic pain; DIE, deep infiltrating endometriosis; EM, endometriosis; EMP, early menopause; FH, family history; IBD, irritable bowel disease; IBS, irritable bowel syndrome; MIG, migraine; NSD, no significant difference; OMA, ovarian endometrioma; PCOS, polycystic ovary syndrome; PD, psychiatric disorder; PHQ, patient health questionnaire; pts, patients; Ref., reference; SD, standard deviation; SUP, superficial peritoneal endometriosis.

**Table 2:** A summary of studies regarding a higher prevalence of migraine in women with endometriosis.

Ref.	Year	Population	Comparison	Findings	EM Diagnosis
Ferrero et al. [32]	2004	133 EM pts	166 HC	MIG more common in EM pts than in HC (38.3% vs. 15.5%)	histologically proven
Yang et al. [33]	2012	20,220 EM pts, aged 18–51	263,767 non-EM pts aged 18–51	MIG more common in EM pts than HC (OR 1.70, 95% CI 1.59, 1.82)	diagnosed by obstetrics and gynecology physicians (ICD-9 CM codes: 617.x)
Wu et al. [34]	2022	167 EM pts, among those 49 with co-occurring AM	190 pts with other benign gynecological disorders, 41 patients with AM with excluded EM	MIG more common in EM pts than HC (29.9% vs. 12.1%); NSD between isolated AM pts and HC (9.8% vs. 12.1%); higher MIG prevalence in EM with AM than isolated EM pts	EM verified by surgery; AM diagnosed with transvaginal ultrasound or histologically
Karamustafaoglu Balci et al. [35]	2019	185 EM pts	168 non-EM pts	MIG more common in EM than in non-EM pts (44.7% vs. 26.8%)	laparoscopy
Sultana et al. [36]	2024	190 EM pts, aged 18–49	190 non-EM pts, aged 18–49	EM pts had 6.13 times higher odds for MIG than HC	laparoscopy or laparotomy
Al-Jefout et al. [37]	2018	55 EM pts, aged 18–55	~3519 non-EM pts, aged 18–55	MIG more common in EM pts than HC (14.5% vs. 5.4%)	not specified, based on self-administered questionnaire
Swift et al. [38]	2022	410 EM pts, aged 18–55	not specified number of non-EM pts	MIG more common in EM than HC (19.8% vs. 13.2%)	self-reported and pelvic ultrasound data
Tietjen et al. [39]	2006	50 MIG pts, aged 22–50	52 age-matched HC	EM more common in MIG pts than HC (30% vs. 4%)	laparoscopy
Tietjen et al. [40]	2007	171 MIG pts, aged 18–62	104 pts without headaches, aged 22–67	EM more common in MIG pts than HC (22% vs. 9.6%)	laparoscopy

AM, adenomyosis; CI, confidence interval; EM, endometriosis; HC, healthy controls; ICD, International Classification of Diseases; MIG, migraine; NSD, no significant difference; OR, odds ratio; pts—patients; Ref., reference; vs., versus.

**Table 3:** A summary of studies regarding the correlation between different migraine types and endometriosis.

Ref.	Year	Population	Comparison	Findings	EM Diagnosis
Merki-Feld et al. [41]	2024	94 EM+MIG pts, premenopausal aged > 18 years	observational study, identification of MIG phenotypes in pts with MIG and EM	MwA in 41% of study group	biopsy-confirmed EM
Pasquini et al. [42]	2023	70 EM+MIG pts	61 EM pts	pure menstrual MIG in 18.6%; menstrually-related MIG in 45.7%; non-menstrual MIG in 35.7%; dysuria and dysmenorrhea were significantly more frequent in EM+MIG pts than in EM pts without MIG	EM diagnosis based on a surgical evaluation or both imaging and clinical symptoms
Tietjen et al. [40]	2007	171 MIG pts	104 non-MIG pts	EM in 22% of MIG pts and 9.6% of non-MIG pts; chronic headache more frequently in EM+MIG pts compared to pts without EM; EM in 64% of chronic MIG pts compared to 36% of episodic MIG pts	laparoscopy
Spierings and Padamsee [43]	2015	96 MIG pts, A 34.0 ± 8.0 (SD) years	chronic MIG vs. episodic MIG pts	NSD between EM and the incidence of chronic versus episodic MIG	not specified

A, mean age; EM, endometriosis; MIG, migraine; MwA, migraine with aura; NSD, no statistical difference; pts, patients; Ref., reference; SD, standard deviation; vs., versus.

**Table 4:** A summary of studies regarding pain symptoms in patients with endometriosis and migraine.

Ref.	Year	Population	Comparison	Findings	EM Diagnosis
Ghiasi et al. [44]	2024	~971 CPP pts, aged 7–55	~284 pts with none or minimal PP, aged 7–55	MIG more common in PP pts; higher number of pain comorbidities in EM+MIG pts	surgically
Maitrot-Mantelet et al. [45]	2019	192 EM pts, aged 18–42	132 non-EM pts, aged 18–42	higher VAS scores for CPP among EM+MIG pts	histologically proven
Miller et al. [46]	2018	205 adl with EM+MIG; 91 adl with only EM	65 adl without MIG or EM; 30 adl with only MIG	severe MIG related pain (+) correlated with higher odds of EM	surgically
Evans et al. [47]	2018	101 DYSM pts and confirmed EM, aged over 16	22 non-EM DYSM pts, aged over 16	MIG in EM pts (+) correlated with the severity of DYSM	laparoscopy
Neumeier et al. [48]	2023	94 EM+MIG pts, aged 20–53	250 EM only pts, aged 21–56	DYSCH, DYSU, and more severe DYSM onset on menarche more common in EM+MIG pts	biopsy-confirmed
Selntigia et al. [49]	2024	50 EM/AM+MIG pts	100 EM/AM only pts; 100 MIG only pts	more severe and more prevalent EM symptoms in EM+MIG pts than in EM only pts; worse MIG symptoms in EM pts than in only MIG pts	clinical symptoms and transvaginal ultrasound imaging
Spierings and Padamsee [43]	2015	96 MIG pts, A 34.0 ± 8.0 (SD) years	chronic MIG vs. episodic MIG pts	41,2% of chronic MIG compared to 22.2% of episodic MIG in pts with menstrual-cycle disorders; dysmenorrhea in 51% of chronic MIG pts compared to 28.9% of episodic MIG pts;	not specified

(+), positive; adl, adolescents; AM, adenomyosis; CPP, chronic pelvic pain; DYSM, dysmenorrhea; DYSU, dysuria; DYSCH, dyschezia; EM, endometriosis; HC, healthy controls; MIG—migraine; PP, pelvic pain; pts—patients; Ref., reference; VAS, visual analogue scale.

**Table 5:** A summary of studies regarding the putative underlying causes of migraine and endometriosis comorbidity.

Ref.	Year	Population	Comparison	Findings	EM Diagnosis
Sasamoto et al. [50]	2023	142 EM pts, A 18 years	comparison groups based on different pain subtypes among EM pts	different patterns of CGRP levels changes observed in EM+MIG pts than in other subgroups; significant correlation between CGRP levels and MIG or EM pain frequency	laparoscopy
Raffaelli et al. [51]	2021	31 MIG only pts, 30 EM only pts, 30 MIG+EM pts	31 HC, age matched	27 proteins, (+) or (−) correlated with MIG in EM pts; 28 proteins correlated with distal rather than pelvic-localized pain.	histologically proven

(+), positive; (−), negative; adl, adolescents; A, mean age; CGRP—calcitonin gene-related peptide, EM—endometriosis; HC, healthy controls; MIG—migraine; pts—patients; Ref., reference.

**Table 6:** A summary of studies regarding the genetic bases of migraine and endometriosis correlation.

Ref.	Year	Population	Comparison	Findings	EM Diagnosis
McGrath et al. [52]	2023	5432 EM pts and 92,344 controls for the 1st cohort; 2085 EM pts and 52,125 controls for the 2nd cohort	analysis within specific subgroups	GC with EM assessed for 74 traits; 22 traits with a significant (+) GC with EM; 1 locus for MIG with suggestive evidence of having 2 distinct causal variants (PPA4) with EM; GC between MIG and EM: 0.14	1st cohort—both surgically diagnosed and self-reported EM; second cohort—EM cases with an ICD10 diagnostic code, likely surgically confirmed
Nyholt et al. [53]	2009	457 DT and 815 MT with EM and MIG	analysis within specific subgroups	increased correlation in both EM and MIG for MT compared to DT of proband twins suffering from either trait; significant additive GC detected between MIG and EM; significant bivariate heritability: 15.37%	surgically confirmed EM

(+), positive; (−), negative; DT, dizygotic co-twins; EM, endometriosis; GC, genetic correlation; ICD, International Classification of Diseases; MIG, migraine; MT, monozygotic co-twins; pts, patients; Ref., reference.

## Abbreviations

The following abbreviations are used in this manuscript:

ASRM	American Society of Reproductive Medicine
CGRP	Calcitonin gene-related peptide
HIT	Headache Impact Test
ICHD-3	International Classification of Headache Disorders, 3rd edition
PRISMA	Preferred Reporting Items for Systematic Reviews and Meta-Analyses
UK	United Kingdom
USG	Ultrasonography
VAS	Visual analog scale
WHO	World Health Organization

## References

[1] Steiner T.J., Stovner L.J., Vos T. (2016). GBD 2015: Migraine is the third cause of disability in under 50s. J. Headache Pain.

[2] Dong L., Dong W., Jin Y., Jiang Y., Li Z., Yu D. (2025). The Global Burden of Migraine: A 30-Year Trend Review and Future Projections by Age, Sex, Country, and Region. Pain Ther..

[3] (2018). Headache Classification Committee of the International Headache Society (IHS) The International Classification of Headache Disorders, 3rd edition. Cephalalgia.

[4] Ferrari M.D. (1998). Migraine. Lancet.

[5] Fraser C.L., Hepschke J.L., Jenkins B., Prasad S. (2019). Migraine Aura: Pathophysiology, Mimics, and Treatment Options. Semin. Neurol..

[6] Dodick D.W. (2018). A Phase-by-Phase Review of Migraine Pathophysiology. Headache.

[7] Karsan N., Goadsby P.J. (2018). Biological insights from the premonitory symptoms of migraine. Nat. Rev. Neurol..

[8] Kim J.R., Park T.J., Agapova M., Blumenfeld A., Smith J.H., Shah D., Devine B. (2025). Healthcare resource use and costs associated with the misdiagnosis of migraine. Headache.

[9] Blumenfeld A., Dueland A.N., Evers S., Jenkins B., Martelletti P., Sommer K. (2022). Practical Insights on the Identification and Management of Patients with Chronic Migraine. Pain Ther..

[10] WHO (2011). Atlas of Headache Disorders and Resources in the World 2011.

[11] Giudice L.C., Kao L.C. (2004). Endometriosis. Lancet.

[12] Parasar P., Ozcan P., Terry K.L. (2017). Endometriosis: Epidemiology, Diagnosis and Clinical Management. Curr. Obs. Gynecol. Rep..

[13] Fonseca M.A.S., Haro M., Wright K.N., Lin X., Abbasi F., Sun J., Hernandez L., Orr N.L., Hong J., Choi-Kuaea Y. (2023). Single-cell transcriptomic analysis of endometriosis. Nat. Genet..

[14] Foley C.E., Ayers P.G., Lee T.T. (2022). Abdominal Wall Endometriosis. Obs. Gynecol. Clin. North. Am..

[15] Davis A.C., Goldberg J.M. (2017). Extrapelvic Endometriosis. Semin. Reprod. Med..

[16] Nisolle M., Donnez J. (1997). Peritoneal endometriosis, ovarian endometriosis, and adenomyotic nodules of the rectovaginal septum are three different entities. Fertil. Steril..

[17] Garcia Garcia J.M., Vannuzzi V., Donati C., Bernacchioni C., Bruni P., Petraglia F. (2023). Endometriosis: Cellular and Molecular Mechanisms Leading to Fibrosis. Reprod. Sci..

[18] Sinaii N., Plumb K., Cotton L., Lambert A., Kennedy S., Zondervan K., Stratton P. (2008). Differences in characteristics among 1,000 women with endometriosis based on extent of disease. Fertil. Steril..

[19] (1997). Revised American Society for Reproductive Medicine classification of endometriosis: 1996. Fertil. Steril..

[20] Moore J., Copley S., Morris J., Lindsell D., Golding S., Kennedy S. (2002). A systematic review of the accuracy of ultrasound in the diagnosis of endometriosis. Ultrasound Obs. Gynecol..

[21] Fassbender A., Vodolazkaia A., Saunders P., Lebovic D., Waelkens E., De Moor B., D’Hooghe T. (2013). Biomarkers of endometriosis. Fertil. Steril..

[22] Vodolazkaia A., El-Aalamat Y., Popovic D., Mihalyi A., Bossuyt X., Kyama C.M., Fassbender A., Bokor A., Schols D., Huskens D. (2012). Evaluation of a panel of 28 biomarkers for the non-invasive diagnosis of endometriosis. Hum. Reprod..

[23] Ghai V., Jan H., Shakir F., Haines P., Kent A. (2020). Diagnostic delay for superficial and deep endometriosis in the United Kingdom. J. Obs. Gynaecol..

[24] Jenabi E., Khazaei S. (2020). Endometriosis and migraine headache risk: A meta-analysis. Women Health.

[25] van der Vaart J.F., Merki-Feld G.S. (2022). Sex hormone-related polymorphisms in endometriosis and migraine: A narrative review. Womens Health.

[26] Flores-Caldera I., Ramos-Echevarría P.M., Oliveras-Torres J.A., Santos-Piñero N., Rivera-Mudafort E.D., Soto-Soto D.M., Hernández-Colón B., Rivera-Hiraldo L.E., Mas L., Rodríguez-Rabassa M. (2021). Ibero-American Endometriosis Patient Phenome: Demographics, Obstetric-Gynecologic Traits, and Symptomatology. Front. Reprod. Health.

[27] Agarwal S.K., Soliman A.M., Zivkovic M., Zhu X., Pan I. (2020). PDB99 baseline characteristics of endometriosis patients participating in a real world prospective cohort study: An interim analysis of the elagolix longitudinal outcomes (lotus) study. Value Health.

[28] Vannuccini S., Lazzeri L., Orlandini C., Morgante G., Bifulco G., Fagiolini A., Petraglia F. (2018). Mental health, pain symptoms and systemic comorbidities in women with endometriosis: A cross-sectional study. J. Psychosom. Obstet. Gynecol..

[29] Sarria-Santamera A., Yemenkhan Y., Terzic M., Ortega M.A., Asunsolo Del Barco A. (2023). A Novel Classification of Endometriosis Based on Clusters of Comorbidities. Biomedicines.

[30] Metzler J.M., Imesch P., Dietrich H., Knobel C., Portmann L., Neumeier M.S., Merki-Feld G.S. (2024). Impact of family history for endometriosis, migraine, depression and early menopause on endometriosis symptoms, localization and stage: A case control study. Eur. J. Obstet. Gynecol. Reprod. Biol..

[31] Karp B.I., Sinaii N., Nieman L.K., Silberstein S.D., Stratton P. (2011). Migraine in women with chronic pelvic pain with and without endometriosis. Fertil. Steril..

[32] Ferrero S., Pretta S., Bertoldi S., Anserini P., Remorgida V., Del Sette M., Gandolfo C., Ragni N. (2004). Increased frequency of migraine among women with endometriosis. Hum. Reprod..

[33] Yang M.H., Wang P.H., Wang S.J., Sun W.Z., Oyang Y.J., Fuh J.L. (2012). Women with endometriosis are more likely to suffer from migraines: A population-based study. PLoS ONE.

[34] Wu Y., Wang H., Chen S., Lin Y., Xie X., Zhong G., Zhang Q. (2022). Migraine Is More Prevalent in Advanced-Stage Endometriosis, Especially When Co-Occuring with Adenomoysis. Front. Endocrinol..

[35] Karamustafaoglu Balci B., Kabakci Z., Guzey D.Y., Avci B., Guler M., Attar E. (2019). Association between endometriosis, headache, and migraine. J. Endometr. Pelvic Pain Disord..

[36] Sultana S., Chowdhury T.A., Chowdhury T.S., Mahmud N., Sultana R., Mahtab N.T., Sharker Y., Ahmed F. (2024). Migraine among women with endometriosis: A hospital-based case-control study in Bangladesh. AJOG Glob. Rep..

[37] Al-Jefout M., Alawar S., Balayah Z., Alhareb A., Ameri F.A., Alhosani M., Naqbi H.A. (2018). Self-reported prevalence of endometriosis and its symptoms in the United Arab Emirates (UAE). Biomed. Pharmacol. J..

[38] Swift B., Zondervan K., Becker C., Rahmioglu N. (2022). The Cyprus women’s health research (COHERE) initiative: Estimating the prevalence, symptomatology, associated risk factors and economic burden of endometriosis in an Eastern Mediterranean population. Hum. Reprod..

[39] Tietjen G.E., Conway A., Utley C., Gunning W.T., Herial N.A. (2006). Migraine is associated with menorrhagia and endometriosis. Headache.

[40] Tietjen G.E., Bushnell C.D., Herial N.A., Utley C., White L., Hafeez F. (2007). Endometriosis is associated with prevalence of comorbid conditions in migraine. Headache.

[41] Merki-Feld G., Dietrich H., Imesch P., Gantenbein A.R., Sandor P., Schankin C.J. (2024). Investigating migraine phenotype and dynamics in women with endometriosis: An observational pilot study. Acta Neurol. Belg..

[42] Pasquini B., Seravalli V., Vannuccini S., La Torre F., Geppetti P., Iannone L., Benemei S., Petraglia F. (2023). Endometriosis and the diagnosis of different forms of migraine: An association with dysmenorrhoea. Reprod. BioMed. Online.

[43] Spierings E.L., Padamsee A. (2015). Menstrual-Cycle and Menstruation Disorders in Episodic vs Chronic Migraine: An Exploratory Study. Pain Med..

[44] Ghiasi M., Chang C., Shafrir A.L., Vitonis A.F., Sasamoto N., Vazquez A.I., Divasta A.D., Upson K., Sieberg C.B., Terry K.L. (2024). Subgroups of pelvic pain are differentially associated with endometriosis and inflammatory comorbidities: A latent class analysis. Pain.

[45] Maitrot-Mantelet L., Hugon-Rodin J., Vatel M., Marcellin L., Santulli P., Chapron C., Plu-Bureau G. (2020). Migraine in relation with endometriosis phenotypes: Results from a French case-control study. Cephalalgia.

[46] Miller J.A., Missmer S.A., Vitonis A.F., Sarda V., Laufer M.R., DiVasta A.D. (2018). Prevalence of migraines in adolescents with endometriosis. Fertil. Steril..

[47] Evans S.F., Brooks T.A., Esterman A.J., Hull M.L., Rolan P.E. (2018). The comorbidities of dysmenorrhea: A clinical survey comparing symptom profile in women with and without endometriosis. J. Pain Res..

[48] Neumeier M.S., Pohl H., Dietrich H., Knobel C., Portmann L., Metzler J., Imesch P., Merki-Feld G.S. (2023). Endometriosis Features in Women With and Without Migraine. J. Women’s Health.

[49] Selntigia A., Exacoustos C., Ortoleva C., Russo C., Monaco G., Martire F.G., Rizzo G., Della-Morte D., Mercuri N.B., Albanese M. (2024). Correlation between endometriosis and migraine features: Results from a prospective case-control study. Cephalalgia.

[50] Sasamoto N., Ngo L., Vitonis A.F., Dillon S.T., Sieberg C.B., Missmer S.A., Libermann T.A., Terry K.L. (2023). Plasma proteomic profiles of pain subtypes in adolescents and young adults with endometriosis. Hum. Reprod..

[51] Raffaelli B., Overeem L.H., Mecklenburg J., Hofacker M.D., Knoth H., Nowak C.P., Neeb L., Ebert A.D., Sehouli J., Mechsner S. (2021). Plasma calcitonin gene-related peptide (CGRP) in migraine and endometriosis during the menstrual cycle. Ann. Clin. Transl. Neurol..

[52] McGrath I.M., Montgomery G.W., Mortlock S. (2023). Genomic characterisation of the overlap of endometriosis with 76 comorbidities identifies pleiotropic and causal mechanisms underlying disease risk. Hum. Genet..

[53] Nyholt D.R., Gillespie N.G., Merikangas K.R., Treloar S.A., Martin N.G., Montgomery G.W. (2009). Common genetic influences underlie comorbidity of migraine and endometriosis. Genet. Epidemiol..

[54] Borończyk M., Zduńska A., Węgrzynek-Gallina J., Grodzka O., Lasek-Bal A., Domitrz I. (2025). Migraine and stroke: Correlation, coexistence, dependence—A modern perspective. J. Headache Pain.

[55] Koehler P.J., Boes C.J. (2023). History of migraine. Handb. Clin. Neurol..

[56] Haanes K.A., Edvinsson L. (2019). Pathophysiological Mechanisms in Migraine and the Identification of New Therapeutic Targets. CNS Drugs.

[57] Lamceva J., Uljanovs R., Strumfa I. (2023). The Main Theories on the Pathogenesis of Endometriosis. Int. J. Mol. Sci..

[58] Imperiale L., Nisolle M., Noël J.C., Fastrez M. (2023). Three Types of Endometriosis: Pathogenesis, Diagnosis and Treatment. State of the Art. J. Clin. Med..

